# High-throughput recombinant protein expression in *Escherichia coli*: current status and future perspectives

**DOI:** 10.1098/rsob.160196

**Published:** 2016-08-31

**Authors:** Baolei Jia, Che Ok Jeon

**Affiliations:** Department of Life Science, Chung-Ang University, Seoul 06974, Republic of Korea

**Keywords:** high-throughput, recombinant protein expression, *Escherichia coli*, 5′UTR and N-terminal codons, fusion tag, membrane protein

## Abstract

The ease of genetic manipulation, low cost, rapid growth and number of previous studies have made *Escherichia coli* one of the most widely used microorganism species for producing recombinant proteins. In this post-genomic era, challenges remain to rapidly express and purify large numbers of proteins for academic and commercial purposes in a high-throughput manner. In this review, we describe several state-of-the-art approaches that are suitable for the cloning, expression and purification, conducted in parallel, of numerous molecules, and we discuss recent progress related to soluble protein expression, mRNA folding, fusion tags, post-translational modification and production of membrane proteins. Moreover, we address the ongoing efforts to overcome various challenges faced in protein expression in *E. coli*, which could lead to an improvement of the current system from trial and error to a predictable and rational design.

## 1. Introduction

High-throughput studies can be defined as research that allows thousands of concurrent measurements of biological molecules to be obtained and thus makes large-scale repetition feasible. This technology originated in the early 1990s when the first automated DNA sequencers were developed and human genome sequencing was initiated [1]. In the post-genomic era, the use of high-throughput techniques has increased dramatically in terms of measuring DNA, RNA, proteins, lipids and metabolites [2], and these techniques have been successfully applied to answer diverse biological questions related to cancer biology, ecology, cell biology and systems biology [3].

Protein expression and purification play a central role in biochemistry. Recombinant proteins can be expressed using prokaryotic systems (*Escherichia coli* and *Bacillus subtilis*), eukaryotic systems (yeast, insect cells and mammalian cells) or *in vitro* systems. The *E. coli* system is the first-choice host for the initial screening of recombinant protein expression, because these cells can be readily manipulated, are cultured inexpensively and grow rapidly [4,5]. In recent years, numerous new strains, vectors and tags have been developed to overcome the limitations of this system, which include codon bias, inclusion body formation, toxicity, protein inactivity, mRNA instability and lack of post-translational modification [4].

The *E. coli* expression system has been widely examined, but protein expression and purification performed using this system are labour-intensive and time-consuming. Thus, a parallel and high-throughput approach must be employed in protein expression and purification, which has been the bottleneck in studies of protein function, structure and application in the post-genomic era [6]. As high-throughput methods of protein production were proposed at the beginning of this century [7], the techniques have become widely available [8–11], and recombinant proteins in inclusion body forms have even been expressed and purified in a parallel approach [12]. We also developed our own systems for purifying proteins from archaea in parallel [13–15]. Because numerous advances in these methods have been made over the past few years, in this review, we discuss the advantages and disadvantages of the current methods—specifically, those targeting gene cloning, vector construction, fusion tags and host strains.

## 2. High-throughput preparation of target genes

**Figure 1.**
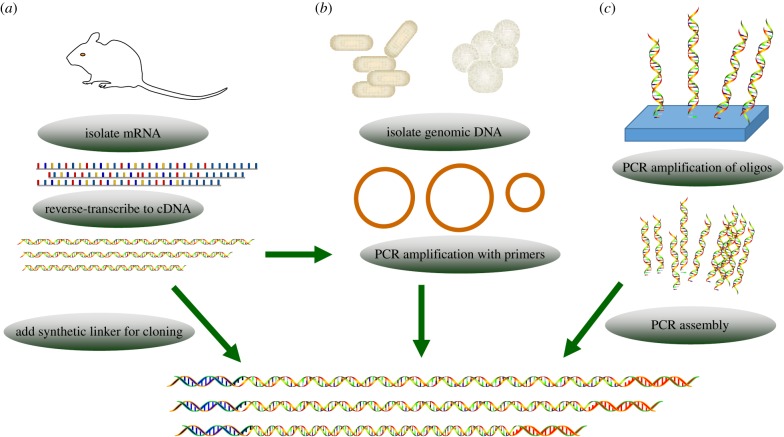
Three strategies for preparing target genes. (*a*) Target genes can be obtained from a cDNA library after reverse transcription. (*b*) PCR can be used to amplify genes from a cDNA library or genomic DNA. (*c*) Array-based gene synthesis through the assembly of short oligos can be used to produce customized genes.

Historically, collections of genes to be expressed have been directly cloned from cDNA libraries as a pool into specific vectors (figure 1*a*) [16]. This method was used by Büssow *et al.*, who constructed a human fetal brain cDNA expression library in *E. coli* in 2000 [17]. The library contained a total of 193 536 clones, but only 37 830 (19.6%) clones expressed proteins. Further investigation revealed that some of the genes were not in the correct reading frame or contained partial coding sequences. Subsequently, a novel human cDNA expression library enabling the selection of open reading frames based on histidine prototrophy was developed in yeast [16]; in this library, approximately 60% of the clones were in the correct open reading frame. However, there are two limitations to the application of expression libraries, most notably in mammalian cells. First, the presence of untranslated regions at both ends of clones makes it challenging to attach fusion tags to either end of the proteins of interest. Second, although the process is laborious, genes of interest must be frequently fished out of a library for use in experiments [18].

Polymerase chain reaction (PCR) is the most widely used technique for obtaining target genes and is invariably the first step in any effort to express recombinant proteins (figure 1*b*). After genes of interest have been selected, a batch of primers can be designed based on the coding sequences using online tools such as PrimerCE [19] and HTP-OligoDesigner [20]. High-throughput PCR and PCR-product purification are now mature technologies that can be completed using automated laboratory workstations [21]. However, problems such as the absence of a band (faint band) in gels, non-specific bands and primer-dimers may occur after PCR and slow the experimental process. These problems can be overcome by adjusting PCR parameters such as annealing temperature and primer concentration or by using the cloning methods discussed below.

Another approach used for obtaining target genes is *de novo* synthesis of DNA (figure 1*c*). Solid-phase (on-column) DNA synthesis involving chemical methods has been traditionally used, but the difficult of synthesis increases with DNA length. Moreover, the synthesis can cost approximately $0.15 per base, and considerably more for high-throughput synthesis. New array-based methods for synthesizing long DNA sequences with increased accuracy have been developed [22–24] and are expected substantially lower the synthesis cost [25]. The main advantage of *de novo* gene synthesis is that researchers can freely design genes of interest without limitations imposed by the use of natural templates [26]. Moreover, the use of codon-optimized genes can ensure reliable expression, increased protein yield and protein solubility [27]. With further developments in the technique, the applicability of *de novo* DNA synthesis to high-throughput assays is expected to increase.

## 3. High-throughput gene-cloning systems

**Figure 2.**
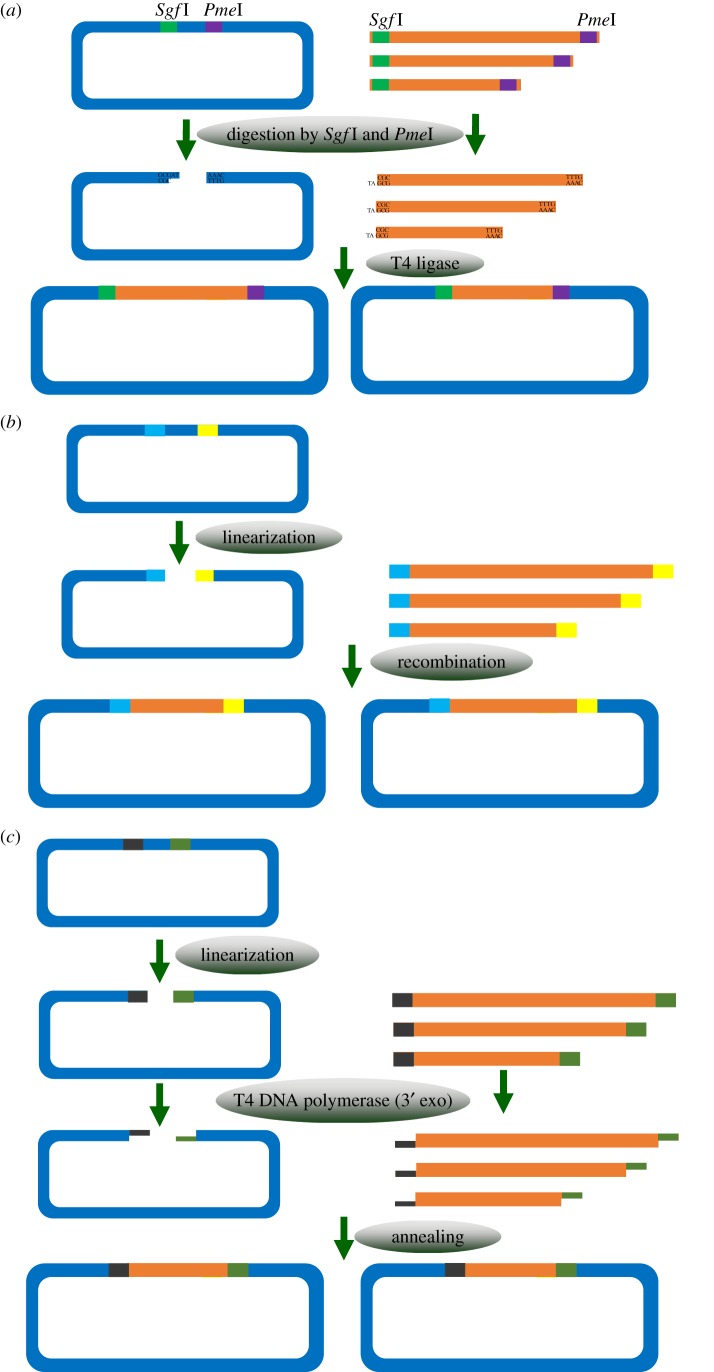
Schematic diagrams and principles of the construction of recombinant expression vectors. Target genes featuring two adapters are obtained from PCR or gene synthesis. (*a*) Construction of expression vectors using restriction enzymes and ligases. The vector and target genes harbouring restriction sites are digested using two rare-cutting enzymes, *Sgf*I and *Pme*I. The linearized expression vector and inserts are ligated using T4 ligase to create the construct. (*b*) Construction of expression clones using recombination-based methods. The target genes are flanked by 15–25 bp recombination sites. Recombinase-mediated recombination between the homologous sites present in the insert and vector generates the final vector. (*c*) Construction of expression clones using LIC methods. Linearized vectors and target genes containing complementary 5′-tails are digested using enzymes possessing exonuclease activity in order to increase the proportion of recessed ends. The overhangs can anneal and are ligated *in vivo* after transformation into *E. coli*.

After obtaining target genes, the next step is high-throughput construction of expression vectors. Various cloning methods have been developed to make the process simple, time-efficient and cost-effective (figure 2). Based on the underlying principle, the methods can be classified as restriction enzyme (RE)-based cloning, recombination-based cloning, and annealing-based or ligation-independent cloning (LIC). The advantages and limitations of these methods have been discussed in previous reviews [18,28,29]. In recent years, vast improvements in these methods have been made. Here, we concentrate on the most basic principles and the latest innovations in the existing methods.

### 3.1. Restriction enzyme-based cloning

RE-based cloning performed with DNA ligation has been used for four decades, but it was previously considered to be unsuitable for high-throughput methods because appropriate and compatible REs must be selected for each cloning procedure [7]. The method has received increased attention since 2006, when *Sgf*I and *Pme*I, the two most rare-cutting REs in the human DNA, were used and the Flexi Cloning system was developed by Promega (Madison, WI, USA). The combination of *Sgf*I and *Pme*I has been suggested to allow the cloning of more than 95% of genes of selected model organisms (figure 2*a*) [30]. The experimental procedure is similar to that use for conventional RE-based cloning: target genes are amplified using primers containing adapter sequences and then digested by two enzymes. The vector is also digested, releasing highly toxic *barnase* gene for lethal selection, which can be used as a marker against the parental vector. Subsequently, the target gene and vector are ligated and transformed into competent cells. Nagase *et al.* [31] used the Flexi Cloning system to produce proteins from 1929 open reading frame clones of human genes, demonstrating that this system can be successfully used in a high-throughput manner.

The Golden Gate method [32] relies on the RE *Bsa*I. This method involves restriction digestion and ligation cycling in one tube, which can greatly increase efficiency. One potential limitation of this method may be the occasional presence of one or several internal *Bsa*I site(s) in the gene of interest. An improvement has been made using *Sap*I with a rarer cut site than that of *Bas*I [33]. Another method termed methylation-assisted tailorable ends rational (MASTER) uses the endonuclease *Msp*JI, which specifically recognizes methylated 4-base-pair (bp) sites. Because this modification avoids cuts on corresponding sites within the fragments amplified by PCR, the MASTER method is more suitable for high-throughput cloning [34]. However, it requires expensive methylated primers and PCR amplification of regions, which may introduce errors in longer regions [35].

RE-based cloning methods may hold greater promise than the original methods, because they will be considerably easier to set up for researchers who continue to use traditional digestion–ligation protocols. With the modification of Flexi Cloning and Golden Gate cloning, RE-based cloning methods are expected to emerge as simple, efficient, universal and cost-effective methods for protein production.

### 3.2. Recombination-based cloning

Recombination-based cloning became widely used following the introduction of three cloning systems: Gateway (Thermo Fisher Scientific, Waltham, MA, USA), Echo Cloning (Thermo Fisher Scientific) and Creator (Clontech, Mountain View, CA, USA). Other commercial kits have also been developed, such as Cold Fusion from System Biosciences (Palo Alto, CA, USA) and CloneEZ from GenScript (Piscataway, NJ, USA). In these systems, a site-specific recombinase is employed to construct the required recombinant vector without using any REs and ligases (figure 2*b*). Gateway may be the most popular recombination-based cloning technology for high-throughput approaches and has been used since the late 1990s. The Gateway cloning system exploits the site-specific recombination system used by bacteriophage λ to shuttle sequences between plasmids bearing flanking-compatible recombination attachment (*att*) sites. Once captured as an entry clone, a DNA fragment can be recombined into a variety of destination vectors, resulting in expression clones that can be used in specific applications. The recombination reactions are driven by two enzyme blends known by their commercial names: BP Clonase and LR Clonase [36]. One of the main advantages of the Gateway method is that once an entry clone has been made, the gene of interest can be easily subcloned into a wide variety of destination vectors using the LR reaction.

However, the general use of recombination methods has been limited by high costs and restrictions in the sequence or hosts [37]. Zhang *et al.* [38] created the Seamless Ligation Cloning Extract (SLiCE) method to assemble DNA fragments into vectors in a single *in vitro* recombination reaction using cell extracts from a modified DH10B *E. coli* strain expressing an optimized λ prophage Red recombination system. Motohashi [39] further modified the method by using several common *RecA*^−^ *E. coli* laboratory strains such as DH5α, JM109, DH10B, XL10-gold and Mach1 T1 with careful harvesting (at late log phase) and lysis (at 4°C). Moreover, the cell extracts can be prepared in a simple buffer containing Triton X-100 rather than the expensive commercial lytic reagent [40,41]. The homemade SLiCE from the laboratory strain JM109 can be used in place of the commercial kit at a cost of approximately $0.003 per reaction [41]. The SLiCE-cloning protocol is a simple, convenient and ultra-low-cost method for performing high-throughput cloning.

### 3.3. Ligation-independent cloning

LIC, developed 26 years ago [42], enables directional cloning of any insert after the generation of DNA fragments containing single-stranded complementary ends. The lack of requirement for REs, ligases or recombinases makes LIC inexpensive and easily adaptable to high-throughput performance. However, LIC still requires enzymes such as T4 DNA polymerase and T5 exonuclease, depending on the protocols used, to generate single-stranded complementary ends in target genes and vector sequences (figure 2*c*). Several effective and convenient methods based on the LIC principle have been developed, including Gibson Assembly from NEB (Ipswich, MA, USA) [43], In-Fusion from Clontech [44], polymerase incomplete primer extension cloning [45], sequence and LIC [46], and overlap extension cloning [47,48]. The Gibson Assembly method [39] uses T5 exonuclease to remove portions of the 5′ ends to generate single-stranded complementary overhangs, which are joined together covalently by fusion DNA polymerase and Taq DNA ligase. In a one-step isothermal *in vitro* reaction at 50°C, the fragments can be assembled into a single circular DNA molecule. Since its introduction 7 years ago, the Gibson Assembly method has become a preferred cloning method. Gibson Assembly allows the insertion of one or more DNA fragments into virtually any position of the linearized vector and does not rely on the presence of restriction sites within a particular sequence to be synthesized or cloned. Advantages of using Gibson Assembly in high-throughput cloning include speed, efficiency, scarless assembly with vector and versatility [49].

The LIC method has been successfully used for high-throughput cloning of genes: 130 genes encoding glycoside hydrolases from 13 different organisms were cloned in parallel using LIC and subjected to protein expression screening in *E. coli* [50]. The method also allowed the automated assembly of more than 600 genes encoding transcription activator-like effector nucleases from *Xanthomonas* species in a single day [51]. Moreover, a three-person team cloned 2125 genes from *Pyrococcus furiosus* in three weeks and obtained at least 80% positive clones in a 96-well-plate cloning format using a modified λ-exonuclease-based LIC method [52].

## 4. Expression vectors for high-throughput protein expression

**Figure 3.**
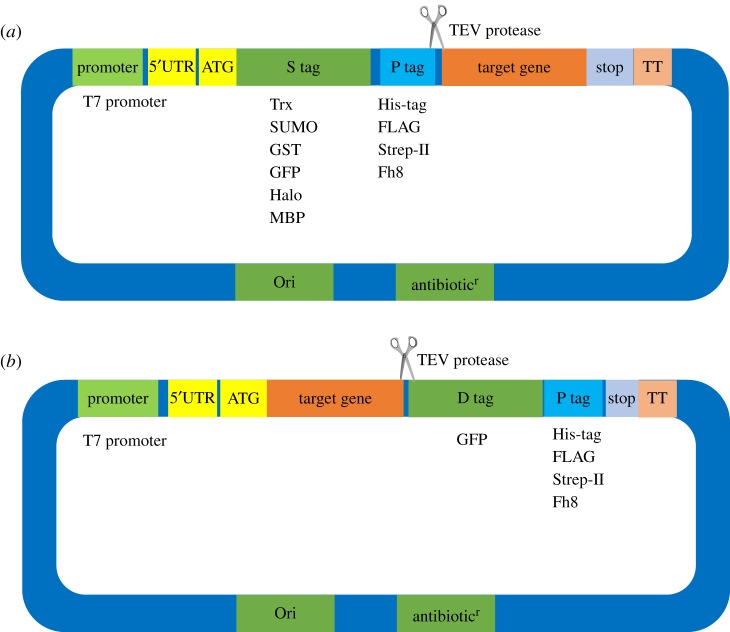
Basic expression vectors for high-throughput expression in *E. coli* of (*a*) cytoplasmic proteins and (*b*) membrane proteins. The T7 promoter is used to control expression of the protein in *E. coli*. The high-throughput assay requires tandem affinity tags, larger tag for protein expression initiation, protein solubility and soluble detection, and smaller tag for purification. TEV protease can be used to remove the tags. The tags for membrane proteins are located at the C-terminus for protein targeting, and GFP is a favourable choice for use as an indicator of protein folding. D tag, detection tag; P tag, purification tag; S tag, solubility and translation initiation tag; TT, transcriptional terminator; 5′UTR, 5′ untranslated region.

An *E. coli* expression vector possesses the same features found in any vector, such as a selection marker (e.g. antibiotic resistance), origin of replication, transcriptional promoter, 5′ untranslated region (5′UTR) and translation initiation site (figure 3). Another critical feature of these expression vectors is the presence of a fusion tag(s) that is transcribed in-frame with the target gene in contrast to the aforementioned elements. Among these various elements, the promoters, 5′UTR, N-terminal codons and fusion tags most strongly affect transcription, protein yields, solubility and purification.

### 4.1. Promoters

An effective promoter for heterologous protein expression in *E. coli* has four key characteristics: first, the promoter is sufficiently strong to allow the accumulation of recombinant protein to greater than or equal to 10–30% of the total cellular proteins; second, it exhibits minimal basal transcriptional activity, and thus unwanted transcription is avoided before induction; third, the promoter enables simple and inexpensive induction; and fourth, promoter activity can be precisely tuned.

The Arabinose promoter and hybrid promoters (trc and tac promoters) are widely used in protein expression. The Arabinose promoter exhibits the lowest basal transcriptional activity, but the efficiency of repression is gene-dependent and the repression level does not always reach zero [53,54]. By contrast, hybrid promoters exhibit leaky expression, and thus these promoters can be problematic for protein expression [55].

**Figure 4.**
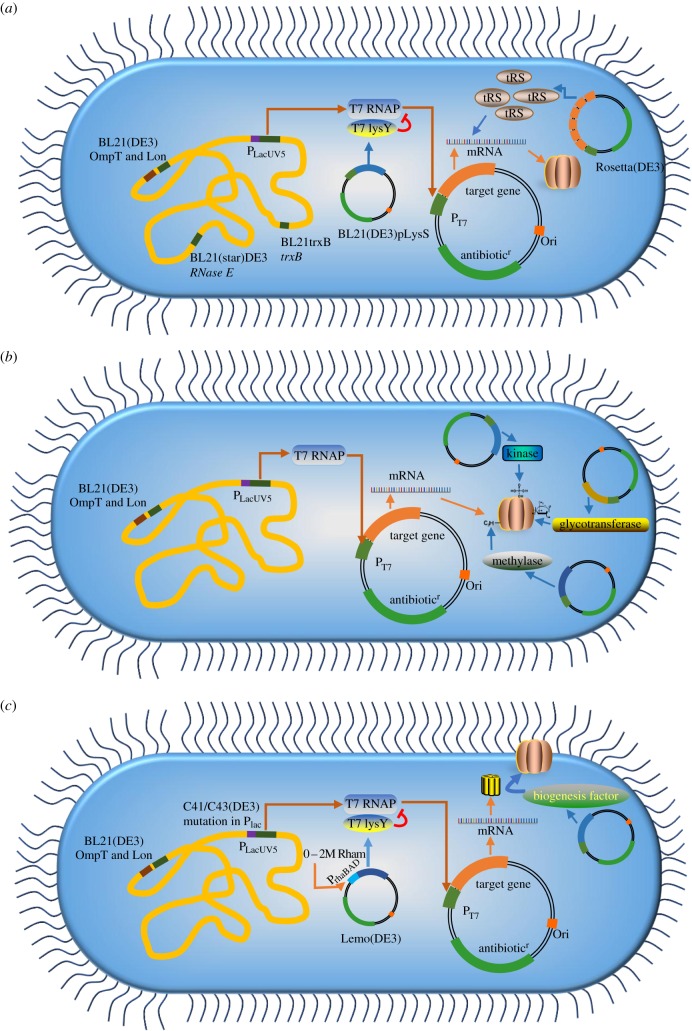
*Escherichia coli* strains for protein expression. (*a*) *Escherichia coli* strains widely used in recombinant protein production. In the expression vector, the target gene is under control of the T7 promoter. In the *E. coli* genome, the gene encoding T7 RNA polymerase is under control of the *lac*UV5. The strain BL21(DE3) is deficient in OmpT and Lon proteases. BL21STAR(DE3) is mutated in RNase E, reducing mRNA degradation. BL21trxB promotes the formation of disulfide bonds. In BL21pLysS(DE3), T7 lysozyme is expressed, and the enzyme inactivates any T7 RNA polymerase that may be produced without induction. Rosetta strains are designed to improve the expression of proteins encoded by genes containing rare codons used in *E. coli*. (*b*) Strategy for expressing a protein with post-translational modification in *E. coli*. Genes encoding kinases, glycosyltransferases, methylases, ligases or other modifying enzymes are coexpressed in order to produce post-translationally modified proteins. (*c*) Overview of *E. coli* strains used in membrane protein production. Walker strains (C41(DE3) and C43(DE3)) are commonly used to overcome the toxicity of membrane proteins. In Lemo21(DE3), expression can be tuned by adding different concentrations of l-rhamnose to the culture. Coexpression of membrane protein biogenesis factors may also facilitate the localization of target proteins. lysY, lysozyme; RNAP, RNA polymerase; tRS, tRNA synthetase.

The Arabinose promoter and hybrid promoters are considered to be strong promoters, but are not as strong as the T7 promoter [56]. The pET expression system featuring the T7 promoter is by far the most widely used system for heterogeneous expression in *E. coli* [57]. T7 promoter activity is strong, and a recombinant protein can accumulate to up to 50% of total cellular proteins [58]. T7 expression hosts such as DE3 strains contain a chromosomal copy of the T7 phage RNA polymerase gene under control of the *lac* promoter derivative *lac*UV5. When isopropyl β-D-1-thiogalactopyranoside (IPTG) is added, LacI binding to the *lac* operator is inhibited, allowing for the expression of T7 polymerase, which transcribes the target gene and leads to recombinant protein production (figures 3 and 4) [59]. Recombinant protein expression can be controlled by coexpressing T7 lysozyme, which inhibits transcription by T7 RNA polymerase [60]. Moreover, previous studies have demonstrated that mutations in the *lac*UV5 promoter can govern the expression of T7 RNA polymerase and lower basal transcription [61]. Tunable expression can be achieved by varying the level of lysozyme produced under the control of the exceptionally well-titratable rhamnose promoter [62]. These advantages make the T7 promoter an attractive choice for the high-throughput production of recombinant proteins.

### 4.2. 5′UTR and N-terminal codons

Gene expression in *E. coli* is influenced by the efficiency of translation, particularly by the initiation step [63]. Both the 5′UTR upstream from the initiation codon and 5′ coding region of a gene transcript are closely related to translation initiation and protein expression [64]. Structural features of the 5′UTR play an important role in controlling translation efficiency, as protein expression is initiated by binding of the ribosome to the Shine–Dalgarno (SD) sequence in the 5′UTR. For example, nucleotide changes to the 5′UTR causing differential formation of mRNA secondary structures can affect protein production levels by up to 600-fold [65]. The spacing and nucleotide sequences between the SD sequence and initiation triplet also have a marked effect on translation efficiency and protein production [66,67]. Optimization of the nucleotide sequences at the junction between the pET vector and coding sequence may enhance protein production [68]. Sequence variants in the region modulate protein expression by as much as 1000-fold; low GC content and relaxed mRNA stability in this region are key, but are not the only factors affecting high expression [68].

Furthermore, the 5′ coding region can also influence translational initiation and gene expression, as the ribosome occupies approximately 15–25 nucleotides on either side of the initiation codon [69,70]. In bacteria, selection pressure favours codons that reduce mRNA folding around the translation start, regardless of whether these codons are frequent or rare [71]. However, rare codons are enriched at the N-terminus of natural genes in most organisms [72,73]. Rare codons at the beginning of genes, which are frequently A/T-rich in the third position in *E. coli*, further correlate with decreased mRNA folding. Using rare codons rather than common codons at the 5′ coding region increases protein expression in *E. coli* by approximately 14-fold (median fourfold) [72]. A recent study further confirmed that the first 18 nucleotides in the coding sequence strongly influence expression based on a study of the expression of 6348 genes from diverse phylogenetic sources. In this region, A and G increase and reduce the probability of high expression, respectively, whereas C and U have intermediate effects [74]. A model based on these experiments indicated that the influential mRNA-folding effects are restricted to the initial approximately 16 codons and that five genes designed by maximizing the folding energy (minimizing folding stability) in the 5′ coding region showed uniformly high expression [74].

To decrease the propensity by the mRNA around the ribosome binding site to form secondary structures, optimization of the AT-content of N-terminal codons has been demonstrated to be a useful strategy, which was used to promote the overexpression of several proteins from bacteria [75], plants [76] and mammals [77] in *E. coli*. Moreover, computational tools have been developed to estimate protein expression and design optimal sequences, such as ExEnSo (Expression Enhancer Software) [78], RBS Calculator [79], RBS Designer [80], UTR Designer [81] and EMOPEC [82]. All calculators were designed for use with *E. coli* and have been shown to give good approximations of protein expression levels [83].

### 4.3. Fusion tags

A prerequisite for high-throughput purification is the addition of a fusion tag at the N- or C-terminus of recombinant proteins. An optimal fusion tag must fulfil these criteria: the tag must enable (i) easy detection of protein expression, (ii) high protein expression and solubility, and (iii) easy isolation of highly pure proteins from *E. coli*. The tags used in early studies were all large proteins, such as Protein A (280 amino acids (aa)) and LacZ (1024 aa) [84,85]. A wide range of tags have been developed [85–87], and the general features of the commonly used tags are listed in table 1. Because the strategies used for expressing cytoplasmic and membrane proteins in *E. coli* differ considerably, we discuss the tags used for these proteins individually below.

**Table 1.** Main characteristics of commonly used fusion tags for high-throughput protein production.

tag	length/size (kDa)	matrix/elution	typical uses	comments	references
His-tag	2–10, typically 6 (0.84)	divalent metal ion (Ni, Co, Cu, Zn)/imidazole or low pH	purification and detection	most common purification tag; denaturing purification possible; rarely affects the structure or function of fusion proteins; an anti-His antibody can be used for detection	[88]
FLAG	8 (1.0)	FLAG antibody/low pH, EDTA or FLAG peptide	purification and detection	small size and high solubility; the presence of an internal enterokinase cleavage site; very expensive resins with limited re-use cycles	[89]
Strep-II	8 (1.1)	Strep-Tactin/biotin or desthiobiotin	purification and detection	short, biologically inert and proteolytically stable; does not interfere with membrane translocation or protein folding	[90]
Fh8	69 (8.0)	Ca^2+^-dependent hydrophobic interaction/ EDTA	purification, increased solubility and expression	relatively low molecular weight; with the combined features of enhancing protein solubility and purification	[86]
Trx	109 (11.7)	phenylarsinine oxide/thiol containing reducing agents	purification and increased solubility	one of the best N-terminal protein fusions to promote soluble expression; purification must be conducted in absence of thiol containing reducing agents until elution step; large tag or elution conditions may affect properties of fusion protein	[91]
SUMO	100 (12.0)	an affinity tag must be added (typically His-tag)	increased solubility and expression	has all the advantages of Trx; SUMO protease efficiently cleaves the tag; enhances membrane proteins expression	[92]
GST	211 (26.0)	glutathione/reduced glutathione	purification, detection and increased expression and solubility	very common purification tag; one-step purification of relatively pure protein; denaturing purification impossible	[85]
GFP	238 (26.9)		detection, increased solubility and expression	native detection protein solubility and expression without antibody, particularly for membrane proteins	[93]
HaloTag	312 (34.0)	Chloroalkane/HaloTag buffer and TEV protease	purification, increased solubility and expression	allow for *in vivo* labelling; functions quickly and results in a highly pure, tag-free protein; cleavage of the tag may result in aggregation of proteins	[85]
MBP	396 (42.0)	cross-linked amylose/maltose	purification, detection, increased expression and solubility	can alleviate toxicity of fusion proteins; the target protein is prone to aggregation after removing tag; the large tag size may affect fusion protein properties and cause immunogenicity	[87]

#### 4.3.1. Fusion tags for cytoplasmic proteins

Fusion tags are invariably introduced at the N-terminus of cytoplasmic proteins, which can provide a reliable context for efficient translation initiation (figure 3*a* and table 1). [86]. The polyhistidine affinity tag, also known as the 6×His-tag, His6 tag and/or hexa-histidine tag, typically consists of six consecutive histidine residues that can bind to several types of immobilized ions (such as nickel, cobalt and copper) [88]. Recombinant galactose dehydrogenase fused with a His-tag was the first protein purified using immobilized metal affinity chromatography [94]. The His-tag is one of most ubiquitously used purification tags, and highly pure protein (more than 80%) can be obtained in a single chromatographic step from *E. coli* together with high expression. The FLAG tag (8 aa) [89] and Strep-II tag (8 aa) [90] are also small tags, but the purification costs may be higher compared with the His-tag. The benefit of adding small fusion tags with minimal charge is that the effects of the tags on recombinant protein structure, activity and characteristics are minimized; however, the recombinant proteins may readily form inclusion bodies [87].

Because the soluble expression and the expression certain non-expressed targets in *E. coli* represent a major bottleneck in protein production, studies continue to develop additional fusion tags for enhancing protein solubility and expression. Large fusion tags positively influence protein solubility and expression efficiency. Thioredoxin (Trx), small ubiquitin-like modifier (SUMO), glutathione S-transferase (GST), green fluorescent protein (GFP), HaloTag and maltose binding protein (MBP), which range in size from 100 to 495 aa, have been widely reported to increase protein expression and solubility [87,91,95–98]. However, the immunogenicity of the tags and their effect on the structure and function of recombinant proteins are major limitations compared with the use of small fusion tags. Another limitation of many of these fusion tags is that they do not function equally well with all target proteins [98]. Recently, an Fh8 tag system (Hitag) with small size (8 kDa) was reported as a robust fusion partner that enables both soluble protein production and the purification of several proteins rapidly and cost-effectively [99].

To overcome the problems associated with different tags, tandem affinity purification (TAP), which involves the use of two affinity tags attached to a target protein, is now commonly used in recombinant protein production. TAP offers an effective and highly specific method for purifying target proteins. After two successive affinity chromatography purifications, the target protein is sufficiently pure for biochemical research. For example, the use of a tandem (His)_6_-calmodulin fusion tag, which combines metal affinity chromatography and hydrophobic interaction chromatography, resulted in the production of eGFP and human p53 that were more than 97% pure after the (His)_6_-calmodulin-tag was cleaved at a thrombin recognition site [100]. Because this technique has been widely exploited, various tags based on other types of TAP have been developed. However, the traditional His-tag, FLAG tag and Strep-II tag remain favourable candidates for use as TAP-tag components [101].

#### 4.3.2. Fusion tags for membrane proteins

Investigation of the structure and function of membrane proteins is challenging because of the difficulties associated with purifying large amounts of these proteins. One difficulty is that membrane proteins must insert into the cytoplasmic membrane and fold properly. To obtain membrane proteins in the folded form, both fusion tags and *E. coli* strains must be designed to be optimal for the membrane protein production process. In membrane proteins, the first hydrophobic transmembrane segment provides the required signal for membrane targeting and insertion [102]; thus, fusion tags are routinely attached to the C-terminus rather than the N-terminus of a target membrane protein, and then the tags are used to monitor the localization, quantity, quality and purification of the membrane protein (figure 3*b*).

One commonly used approach is to fuse a membrane protein to GFP in order to track protein expression, partly because GFP becomes fluorescent only if the upstream target membrane protein integrates into the membrane (table 1) [93,103]. Moreover, GFP fluorescence can be used to rapidly, accurately and easily measure protein expression both in liquid cultures and standard SDS gels [104]. Furthermore, once protein expression has been optimized, the fluorescence from GFP can considerably accelerate detergent screening and purification [105]. However, GFP fusion proteins present certain notable disadvantages; for example, they generate false-positives and protein aggregation occurs after GFP cleavage. Thus, a fluorescent probe that interacts with small His-tag-fused membrane proteins was recently developed; using this probe, target proteins were detected sensitively to 0.02 mg l^−1^ in crude lysates [106].

Whether a given recombinant membrane protein will become localized to the cell membrane or inclusion bodies cannot be predicted. Therefore, additional fusion partners have been developed to facilitate the targeting of membrane proteins to the lipid bilayer. The adenovirus-receptor immunoglobulin variable-type domains were successfully overexpressed as fusions with a set of short, non-globular, negatively charged peptides [107]. Mistic, a short and non-globular *B. subtilis* integral-membrane protein, has been used as a fusion tag for the high-level production of various membrane proteins in their native conformations, including several eukaryotic proteins that are toxic to *E. coli*. [108]. Leviatan *et al.* [109] reported that YaiN and YbeL, two short hydrophilic bacterial proteins, fused to the ends of membrane proteins may facilitate proper folding.

#### 4.3.3. Detection of protein expression using fusion tags

Fusion tags can also be used in protein expression screening, which is essential for obtaining well-expressed and functional proteins. If a His-tag is attached to a target protein, an anti-His antibody can be used to detect the expression and solubility of the recombinant protein in a 96-well format [110]. Proteins can also be labelled with GFP. Here, inclusion body formation leads to the misfolding of GFP and thus a loss of its fluorescence, but if the fusion protein is folded properly, GFP can be synthesized in a fluorescent form. Alternatively, a fluorescent amino acid derivative, BODIPY-FL-lysine, can be translationally incorporated into target proteins; these specifically labelled proteins in cell lysates can be detected using a fluorescence detector [96]. A previous study also reported the fusion of another coloured protein, photoactive yellow protein (or its miniaturized version), to a target protein. In this case, the addition of a precursor of the chromophore to the coexpressed photoactive yellow protein causes a yellow colour to appear; this colour development not only allows target protein expression to be monitored through visual inspection within a few seconds, but also enables protein concentration and purity to be quantified using a spectrometer within a few minutes [111].

#### 4.3.4. Fusion tags and inclusion bodies

Inclusion body formation is a commonly encountered problem, and to promote the solubility of target proteins, high-molecular-weight N-terminal tags such as MBP and GST can be used [97,98]. The soluble expression of recalcitrant proteins can be also improved by designing variants with more favourable native-state energy. Up to five variants encoding from 9 to 67 mutations relative to wild-type can be designed by using the PROSS webserver. The tested variants show higher soluble expression and stability with no change in enzymatic function [112].

However, inclusion body formation does not mean that protein production has failed. The advantages of inclusion bodies are that they (i) produce proteins that are toxic to host cells, (ii) generally allow a high level of expression, and (iii) can be readily separated from bacterial cytoplasmic proteins through centrifugation. The most commonly used methods for refolding inclusion body proteins involve dialysis and on-column folding. Yuan *et al.* [113] reported the continuous-flow mode of a vortex fluid device that enabled parallel processing of protein refolding, and substantially shortened purification times, lowered costs and decreased structure waste streams associated with protein expression. High-throughput inclusion body purification can also be performed using a robotic microfuge: key mutants of RNA polymerase from *Sulfolobus shibatae* are predominantly expressed in an insoluble form, and hundreds of mutants can be automatically purified without the use of tags because inclusion bodies can be readily separated from soluble proteins through centrifugation [12].

#### 4.3.5. Removal of fusion tags

Because many of the aforementioned tags are large polypeptides and may affect the structure and function of target proteins, tag removal is frequently necessary. In all expression vectors, a protease cleavage site is engineered between the tag and target protein. Several proteases can be selected to remove the tag, including SUMO protease, enteropeptidase, thrombin, factor Xa, PreScission and tobacco etch virus (TEV) protease. Among these, SUMO protease only cleaves SUMO tags [92], enteropeptidase and thrombin are incompatible with buffers containing reducing agents [114], factor Xa should not be used in the presence of chelating agents because it binds calcium ions [115], and PreScission leaves behind a Gly-Pro dipeptide on the N-terminus of the recombinant protein after digestion [116]. TEV protease is not inhibited by reducing agents, exhibits very high specificity, is inexpensive, and in most cases cleaves recombinant proteins in a manner that leaves the native protein intact [98,114]. Thus, TEV protease shows the greatest number of advantages as an endoprotease for removing affinity tags for high-throughput purposes.

## 5. *Escherichia coli* expression strains and cell culture

The choice of the strains used to express recombinant proteins also plays a major role in protein expression, solubility and yield. A few *E. coli* strains such as BL21 and its derivatives are widely used (figure 4). Different *E. coli* strains facilitate the expression of proteins containing disulfide bonds or those that are encoded by genes containing rare codons and proteins toxic to *E. coli*. Moreover, coexpression with some genes improves the expression of post-translationally modified proteins. To date, several *E. coli* strains that strongly improve membrane protein production have been engineered. The genotypes and characteristics of these strains are summarized in table 2.

**Table 2.** Main characteristics of commonly used expression strains for high-throughput protein production.

strains	genotype	features	references
BL21(DE3)	F^−^ OmpT hsdS_B_(r_B_^−^ m_B_^−^) gal dcm (DE3)	the most common protein expression strain; leaky expression can lead to uninduced expression of potentially toxic proteins	[117]
BL21Star(DE3)	F^−^ OmpT hsdS_B_(r_B_^−^ m_B_^−^) gal dcm *rne131* (DE3)	mRNA levels and RNA stability are increased in the strain; thus, protein expression may be increased	[118]
Origami(DE3)	F^−^ OmpT hsdS_B_(r_B_^−^ m_B_^−^) gal dcm *trxB gor* (DE3)	the *trxB* and *gor* mutations enable cytoplasmic disulfide bond formation and can be combined with a fusion to Trx	[119]
BL21(DE3)pLysS	F^−^ OmpT hsdS_B_(r_B_^−^ m_B_^−^) gal dcm (DE3) [pLysS Cam^r^]	the pLysS plasmid produces T7 lysozyme to reduce basal level expression, which is suitable for expression of toxic genes	[120]
BL21-CodonPlus(DE3)-RIPL	F^−^ OmpT hsdS_B_(r_B_^−^ m_B_^−^) gal dcm (DE3) *endA* Hte [*argU proL* Cam^r^] [*argU ileY leuW* Strep/Spec^r^]	the CodonPlus strains provide additional copies of rare tRNA genes; the RIPL strain carries genes for Arg (AGA and AGG), Ile (AUA), Pro (CCC) and Leu (CUA)	[121]
Rosetta(DE3)	F^−^ OmpT hsdS_B_(r_B_^−^ m_B_^−^) gal dcm (DE3) [pRARE Cam^r^]	the Rosetta strains enhance the expression of proteins that contain codons rarely used in *E. coli*; the Rosetta (DE3) strain carries genes for Arg (AGG, AGA and CGG), Ile (AUA), Leu (CUA), proline (CCC) and glycine (GGA)	[122]
C41(DE3)/C43(DE3)	selected mutants from BL21(DE3)	the strains harbour mutations in *lac*UV5 promoter, which are effective for expressing toxic and membrane proteins	[56]
Lemo21(DE3)	F^−^ OmpT hsdS_B_(r_B_^−^ m_B_^−^) gal dcm (DE3) [pLemo Cam^r^]	the strain allows for tunable expression of difficult clones; for difficult soluble proteins, tuning the expression level may also result in more soluble, properly folded protein	[123]

### 5.1. Routine *Escherichia coli* strains

BL21 and its derivatives are routinely used for recombinant protein production in *E. coli* (figure 4*a* and table 2). These strains are deficient in the proteases Lon and OmpT, which can increase protein stability. The strain BL21(DE3) contains a chromosomal copy of the T7 RNA polymerase gene for simple and efficient expression of genes under control of the T7 promoter [117]. BL21Star(DE3) contains a mutation in *rne*, the gene that encodes RNase E, and thus the use of BL21Star(DE3) increases mRNA stability and protein expression [118,124]. BL21trxB, a derivative of BL21(DE3), harbours a thioredoxin reductase (*trxB*) mutation, and the strain Origami(DE3) contains mutations in both *trxB* and the gene encoding glutathione reductase (*gor*), which markedly enhances disulfide bond formation in the cytoplasm [119]. BL21(DE3)pLysS contains a pLysS plasmid carrying the gene encoding T7 lysozyme; this strain is used to express proteins that are toxic to cells because T7 lysozyme lowers the leaky expression of target genes [120]. BL21-CodonPlus(DE3) strains provide additional copies of rare tRNA genes; for example, BL21-CodonPlus(DE3)-RIPL (contains the largest number of tRNA genes in the BL21-CodonPlus series) carries genes for Arg-, Ile-, Leu- and Pro- tRNAs [121]. The strains Rosetta and Rosetta (DE3) harbour the pRARE plasmid, in which the genes encoding aminoacyl-tRNA synthetases for Arg, Ile, Leu, Pro and Gly are coexpressed [122]. Both the BL21-CodonPlus(DE3) and Rosetta (DE3) strains efficiently promote the expression of genes harbouring rare codons at high frequencies.

### 5.2. Strategies for expressing proteins with post-translational modifications

The major limitation of using *E. coli* for protein expression is thought to be its lack of available machinery for post-translational modifications. Coexpression of factors that promote post-translational modification appears to be a promising approach for solving this problem (figure 4*b*) [125]. Reversible protein phosphorylation is one of the most important and well-studied post-translational modifications. In *E. coli*, phosphorylation of a target molecule (a mouse or human protein) has been achieved by coexpression with human Jun N-terminal kinase 1 [126]. Protein glycosylation is another major post-translational modification that substantially affects protein stability, distribution and function. The discovery of *N*-linked protein glycosylation in *Campylobacter jejuni* and the functional transfer of this glycosylation system into *E. coli* enabled the production of recombinant glycoproteins in bacteria, although bacterial *N*-glycans structurally differ from their eukaryotic counterparts [127]. Glycoconjugated vaccines can be produced in *E. coli* using this strategy [128]. Furthermore, bacterial *N*-linked glycosylation occurs on scFv antibody fragments and improves the biophysical properties [129]. Ubiquitin is an 8 kDa polypeptide (76 aa) that can be appended to a lysine in target proteins. In *E. coli*, recombinant proteins can be ubiquitinated by co-overexpressing the target protein, ubiquitin and ubiquitin ligases [130]. Additionally, methylation, myristoylation and acetylation have been successfully performed in *E. coli* by coexpressing a methyltransferase, myristoyltransferase and acetylase, respectively [131–133]. Therefore, target proteins can be post-translationally modified in *E. coli* expression systems by coexpressing genes related to the modifications of interest.

### 5.3. *Escherichia coli* strains for expression of membrane proteins

Several recombinant membrane proteins exhibit toxicity upon induction in *E. coli*, and thus only low yields of the properly folded forms of these proteins are obtained [134]. Understanding the physiological response of *E. coli* to recombinant membrane proteins is crucial for identifying bottlenecks in expression and folding [135]. Most of the targeting and translocation of membrane proteins occur through a universally conserved signal-recognition particle (SRP)/secretory (Sec) pathway [136]. Ribosome nascent chain-SRP complexes contact the SRP receptor FtsY at the membrane and thus mediate the transfer of the nascent chain to the Sec translocon. Transfer of the complex into the Sec pore is driven by SecA and ATP hydrolysis. The SecDFYajC complex also plays a critical role in the biogenesis, translocation and folding of membrane proteins [137].

Saturation of the translocon pathway during membrane protein overexpression may cause the accumulation of cytoplasmic aggregates and broad perturbations in the proteome [134]. Two strategies for solving this problem have been employed: (i) tuning of transcription and translation rates and (ii) coexpression of biogenesis factors (figure 4*c*). The strains C41(DE3) and C43(DE3), which are also known as the Walker strains, are BL21(DE3) derivatives harbouring mutations in the *lac*UV5 promoter, influencing the expression levels of T7 RNA polymerase (table 2). A mutation in the lac repressor LacI was also demonstrated to be crucial for favouring tolerance to membrane protein overexpression [138]. Subsequent production of comparatively lower amounts of target proteins in the Walker strains ensured that the Sec translocon was not saturated by the produced proteins [61,139]. Lemo21(DE3) is tunable for membrane protein overexpression, and the amount of membrane protein produced can be readily regulated by exploiting the Sec-translocon capacity of *E. coli* [123]. In Lemo21(DE3), the activity of T7 RNA polymerase can be precisely regulated by expressing T7 lysozyme under control of the l-rhamnose promoter and then modulating the target protein level by adding 0–2 mM l-rhamnose to the culture (table 2) [123].

A complementary approach to lowering protein expression involves increasing the amount of protein biogenesis machinery. Coexpression of the cytoplasmic DnaK/J chaperone system, which functions in protein targeting and folding, improved the production of the magnesium transporter CorA [140]. Moreover, coproduction of the protease FtsH, a membrane-bound quality-control factor, markedly enhanced the yields of G-protein coupled receptors [141]. However, most efforts employing this strategy have not been successful. For example, coexpression of membrane protein biogenesis factors (SRP/FtsY, SecA) and other factors with CorA or G-protein coupled receptors did not improve target protein production [140,141].

Previous studies have also used strategies involving either increasing the expression of factors that enhance membrane protein yields or deleting factors that limit protein production [142,143]. Our understanding of how membrane proteins are translocated and folded in *E. coli* is highly limited, and it appears that the optimal strain for membrane protein production is protein-specific [144]. Currently, C41(DE3), C43(DE3) and Lemo21(DE3) remain the first-choice strains for membrane protein expression.

### 5.4. Culture of *Escherichia coli*

Both culture media composition and culture conditions are important for protein expression. Luria broth (LB) medium is easy to make and is the most commonly used medium for culturing *E. coli*. However, *E. coli* growth in LB stops at a relatively low density, because it contains low amounts of carbohydrates and divalent cations [145]. The 2× yeast extract tryptone, terrific broth and super broth media can also be used and have been shown to be superior to LB for reaching higher cell densities [146]. As cell density increases, oxygen may limit *E. coli* growth and protein expression in batch culture [147]; additional agitation can be generated by using high shaking speeds, shaking in a baffled flask and oxygen-enriched air or pure oxygen [148]. It is also possible to avoid the formation of inclusion bodies by optimizing cell culture conditions. Protein expression in *E. coli* at 15–25°C is commonly induced to increase the solubility of recombinant proteins, and the induction temperature can be lowered to 6–10°C [149]. Uncontrolled pH culture conditions favour recombinant protein aggregation, but stable pH can be maintained by using buffers or through the automatic addition of base or acid [150]. The addition of the cofactors or binding partners required for protein folding to the cultivation media will enhance protein solubility and prevent inclusion body formation [151,152]. Alternatively, the addition of a mild detergent such as Triton X-100 in shaker flasks can enhance the solubility and secretion ratio of aggregation-prone protein [153]. In conclusion, media composition and culture conditions are critical factors for optimizing the expression of recombinant proteins. Although this is attained mostly by trial and error, it may be beneficial.

In contrast to the IPTG induction method, autoinduction was introduced as a convenient method for producing recombinant proteins without inducer addition at the small laboratory scale for *lac* operon-controlled expression systems [146]. Autoinduction medium contains glycerol, lactose and glucose at optimized levels, with glycerol used as the carbon source. Lactose is metabolized for autoinduction once glucose is depleted [154]. Thus, there is no need to monitor the growth, minimizing operator intervention from inoculation to cell harvest, which is preferable in high-throughput experiments. Additionally, there is tighter control of protein induction, improving the expression of toxic proteins. Another advantage of autoinduction is that the medium allows cultures to reach high cell densities and generally produces a greater proportion of soluble target proteins than IPTG-induced expression [155,156]. A disadvantage of autoinduction is that the medium is adversely affected by the aeration level. This can be overcome by using a glucose fed-batch medium, which attenuates oxygen-sensitivity and provides robust high-yield expression under high aeration rates [157]. In some cases, the use of autoinduction medium may not be optimal and is often replaced by other media and induction with IPTG to obtain better yields [158].

The simplest way to grow *E. coli* is batch cultivation, but control of the growth during this process is limited. High-throughput cultivation has undergone rapid evolution in recent years in reducing culture volume, applying in-process real-time monitoring or control at the micro scale, and realizing full automation of the systems [159,160]. A number of emerging cultivation platforms has been commercialized, including microtitre plate culture, micro scale bioreactors and in-parallel fermentation systems [160]. These platforms that significantly reduce culture volume have been adopted extensively to replace shaker flasks [161]. High-throughput cultivation technology, which enables researchers to handle a large number of samples under a range of fermentation conditions in a high-throughput format, can remarkably shorten the timeline from DNA to large-scale protein production [160].

## 6. High-throughput robotic platform for protein expression and purification

High-throughput platforms that can rapidly clone genes, pick colonies, isolate plasmid DNA, transform bacteria, and express and purify proteins have provided opportunities for executing complex molecular biological procedures with little human labour and minimal error rates. Several commercial robotic workstations are available for various purposes, including Equator GX8 Dispenser from Labcyte (Sunnyvale, CA, USA), MicroSys from Genomic Solutions (Ann Arbor, MI, USA), sciFLEXARRAYER dispenser from Scienion (Berlin, Germany) and other systems [162]. These platforms have been used to isolate plasmid DNA, transform bacteria, pick colonies and screen for protein expression [162,163], and a video showing the operation procedure for automatic protein purification is available [164]. Automatic platforms can cost hundreds of thousands of dollars and require routine maintenance, and organizations commonly hire specialists to care for these automated platforms. Thus, if a protein production process does not include adequate numbers of samples to justify this level of spending, it may be prudent to continue to use a manual approach in parallel [165].

## 7. Conclusion and perspectives

Successful recombinant protein expression and purification is frequently indispensable for both basic research studies and biotechnological and commercial applications [166]. High-throughput protein expression and purification in *E. coli* has begun to revolutionize the manner in which studies are conducted in various research fields. Experiments that were typically performed manually to address one protein at a time over a period of several weeks can now be conducted for hundreds of proteins in as little as one week. However, limitations still exist and further improvements are possible.

In terms of obtaining target genes, *in silico* design followed by array-based *de novo* synthesis rather than PCR may become widely used in the future. The major challenges associated with *de novo* synthesis are sequence errors, availability and cost. However, if array-based gene synthesis can be commercialized, the costs could decrease by 3–5 orders of magnitude to 10^3^–10^5^ bp per dollar [25].

Cloning methods have seen rapid advances, and cloning systems used in both commercial and academic settings can be operated with high efficiency, fidelity and reliability, and at a reasonably low cost. The first requirement is to develop a highly flexible expression vector that is fully compatible with high-throughput procedures. An optimal vector must contain a strong but tunable promoter and tags with optimized N-terminal codons to facilitate protein expression, solubility and purification. Large N-terminal tags have been used to enhance translational initiation and promote solubility. However, the cleavage of these large tags may complicate the experiment being conducted and substantially add to the final cost compared to the use of short tags. Given that the downstream costs of testing the functions of individual proteins are often far higher than protein production costs, the cost will probably not dramatically affect experimental workflows. Moreover, new tags are being developed, but considerable room for improvement remains.

Currently, certain post-translational modifications can be achieved in *E. coli* by coexpressing the corresponding enzymes. However, such coexpression invariably affects the growth rate of *E. coli*, and several vectors cannot be readily coexpressed in a single strain. One solution is to integrate genes encoding post-translational modification factors into the genome to create ‘eukaryotic-like’ *E. coli*. Moreover, according to previous studies, tuning or precisely controlling the transcript levels of target proteins is critical for expressing membrane proteins. Membrane protein production is not always successful when the strategy involves coexpressing proteins that function in membrane protein biogenesis. Thus, it is crucial to understand the protein biogenesis mechanism and the physiological response of *E. coli* to membrane protein production. The combination of physiological, genetic and ‘omics’ technologies has improved the understanding of the biogenesis process and has provided rationale for the forward engineering of expression hosts.

Finally, robotic platforms for protein expression and purification are available but are too expensive for most laboratories. However, the protocols and systems currently in use provide an approach required for the cloning, expression and purification of hundreds of proteins in parallel within a few days. The limitations of the protein production process are nearly impossible to solve in a simple and global manner, cases of failure are rarely reported and experience gained does not effectively help guide subsequent efforts. Therefore, a searchable protein expression database that includes strains, vectors, tags, promoters, and cases of success and failure to guide the journey from trial and error towards rational design would be more beneficial to the scientific community than a robotic platform.
